# Influence of Handprint Culture Training on Compliance of Healthcare Workers with Hand Hygiene

**DOI:** 10.1155/2018/3727521

**Published:** 2018-03-05

**Authors:** Hala Fouad, Mona M. A. Halim, HebatAllah F. Algebaly, Nardeen A. Elmallakh

**Affiliations:** ^1^Department of Pediatrics, Kasr Alainy School of Medicine, Cairo University, Cairo, Egypt; ^2^Department of Clinical and Chemical Pathology, Kasr Alainy School of Medicine, Cairo University, Cairo, Egypt

## Abstract

**Objective:**

We aimed to study the effect of visual observation of bacterial growth from handprints on healthcare workers' (HCWs) compliance with hand hygiene (HH).

**Settings:**

Medical and postoperative cardiac surgery units.

**Design:**

Prospective cohort study.

**Subject:**

The study included 40 HCWs.

**Intervention:**

Each HCW was interviewed on 3 separate occasions. The 1st interview was held to obtain a handprint culture before and after a session demonstrating the 7 steps of HH using alcohol-based hand rub, allowing comparison of results before and after HH. A 2nd interview was held 6 weeks later to obtain handprint culture after HH. A 3rd interview was held to obtain a handprint culture before HH. One month before implementation of handprint cultures and during the 12-week study period, monitoring of HCWs for compliance with HH was observed by 2 independent observers.

**Main Results:**

There was a significant improvement in HH compliance following handprint culture interview (*p* < 0.001). The frequency of positive cultures, obtained from patients with suspected healthcare-associated infections, significantly declined (blood cultures: *p* = 0.001; wound cultures:* p* = 0,003; sputum cultures: *p* = 0.005).

**Conclusion:**

The visual message of handprint bacterial growth before and after HH seems an effective method to improve HH compliance.

## 1. Introduction

Annually about hundreds of millions of patients suffer from healthcare-associated infections HCAI worldwide [1]. One of the priorities of the patient safety goals is “First, do no harm” and to reduce the adverse health and social consequences of unsafe healthcare. The World Health Organization (WHO) contributes to this effort through the Patient Safety Programme with its First Global Patient Safety Challenge “Clean Care is Safer Care” (CCiSC), launched in 2005 and dedicated to the prevention of HCAI. One of the important recommendations for reducing HCAI is compliance with hand hygiene practices [2]. The CDC has published a guideline, interactive training and educational materials, and posters for HH compliance [3]. The interactive tools include a set of PowerPoint slides and speaker notes that provide background information on the importance of HH, indications on when to use HH practices and how to properly clean ones' hands, and educational/motivational programs [4]. Successful HH educational program has several key features: knowledge of healthcare workers' (HCWs) perceived importance of HH, monitoring and feedback of HH practices, practical education tools, role-modeling by senior staff, and supportive infrastructure and management [5].

Monitoring HCWs' compliance with HH practices is vital for evaluating whether interventions are successful. WHO recommends using a validated methodology for training observers to directly monitor HH using “My Five Moments for HH” [1]. Other methods for monitoring include patient observations, measuring of HH product consumption (either by volume of product used or through electronic counting devices), and electronic HH compliance monitoring systems [6]. Awareness of being watched can affect the usual behavior of individuals in many unpredictable ways other than simple work productivity effect. In the presence of auditing, HCWs might avoid activities that require HH. Measuring HH product use may overcome avoidance tactics. It is cheaper and generates continuous data to assess compliance of all clinicians without influencing patient care. Disadvantages include overestimation of product use through spillage, wastage, or use by visitors and nonclinical staff entering patient care areas. Electronic devices may overcome the Hawthorne and avoidance effects but are costly and are not widely used outside research studies [7].

We face the challenge of poor compliance with HH among HCWs despite posters, direct observation, and camera monitoring. We conducted this work to study the influence of visualization of bacterial growth from handprints, before and after use of alcohol-based disinfectant, on HCWs' compliance with HH.

## 2. Methodology

The study included 40 HCWs who were recruited from one medical and one postoperative cardiac surgical pediatric intensive care unit (PICU) at Cairo University Pediatric Hospital. We conducted an interventional program about the significance of alcohol-based HH in the two PICUs. The program was carried out in the following steps after* this study was reviewed and approved by the local IRB*.

HCWs were instructed to print both hands with no visible soiling on agar plates (15 × 15 cm) for 5 seconds (Hand Print 0-Before HH) [8] and the plate was then incubated immediately.

**Figure 1:**
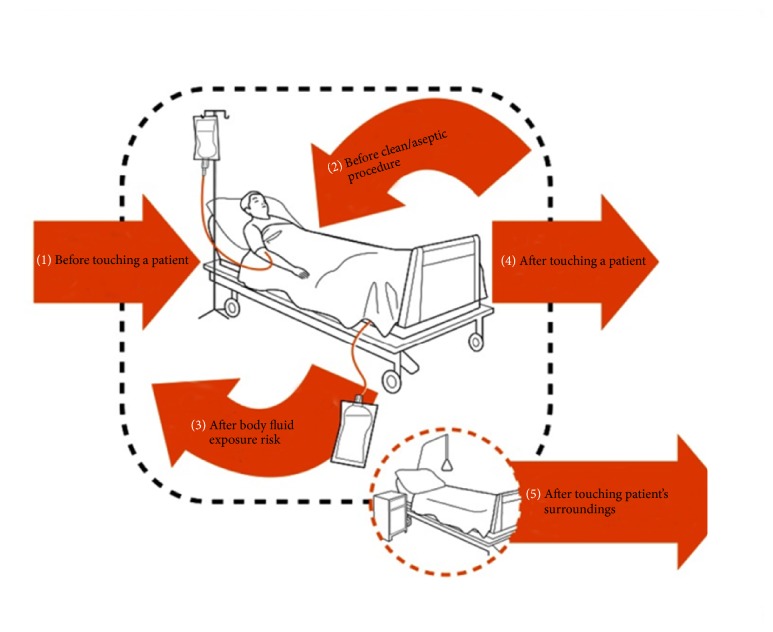
“My Five Moments for Hand Hygiene,” WHO, 2009.

The 7 steps of proper HH technique were demonstrated by a mentor physician from the PICU and infection control team, using alcohol-based liquid hand disinfectant and providing advice on the occasions for HH “My Five Moments for HH” (Figure 1) [1].

Each HCW was monitored while performing proper HH.

A second handprint on agar plate was obtained after proper HH, just after drying of hands (Hand Print 0-After HH).

**Figure 2:**
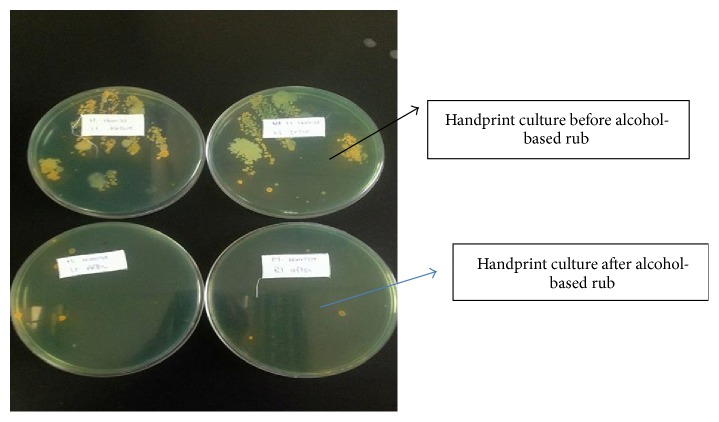
The handprints of one of HCWs before and after hand hygiene by alcohol-based disinfectant.

The HCWs were interviewed 48 hours later to provide them with the results of their handprint cultures, before and after HH, by making them inspect the agar plates and discussing the effect of HH on microbial growth on the plates (Figure 2).

Data were collected from each HCW including age, gender, occupation, working hours per week, nurse to patient ratio, dominant hand, and wearing long sleeves or not.

Handprint cultures were obtained from HCWs 6 weeks later after HH was applied (Hand Print-6 weeks-After HH). The HCWs were interviewed 48 hours later about the results of their handprint culture.

After another 6 weeks, handprint cultures were obtained from each HCW without prior HH, with no visible soiling and not immediately following any procedure involving patient contact (Hand Print-12 weeks-Before HH). Again the HCWs were interviewed 48 hours later about the results of their handprint culture.

One month before implementation of handprint cultures and during the 12-week study period, monitoring of HCWs for compliance with HH was observed by 2 independent observers using the WHO checklist for HCWs HH compliance [1].

### 2.1. Sample Processing

After collection, samples were incubated aerobically for 24 hours. After incubation the plates were observed for colony morphology and colony count, with microscopic examination of gram stained films. Isolates, if any, were identified by standard microbiologic techniques, namely: Gram staining, colony characteristics, and biochemical properties, according to [9]. Cefoxitin disc (30 *μ*g) was used to screen methicillin-resistant* Staphylococcus aureus* (MRSA). Transient flora was defined as any pathogen other than coagulase-negative staphylococci (CoNS),* Corynebacterium* sp.,* Micrococcus* sp., or* Bacillus* sp. Gram-negative lactose nonfermenters included* Acinetobacter*,* Bordetella*,* Burkholderia*,* Legionella*,* Moraxella*,* Pseudomonas*, and* Stenotrophomonas*. Gram-negative lactose fermenters included* Enterobacter *spp*., Escherichia coli, *and* Klebsiella*.

### 2.2. Statistical Analysis

Data were analyzed by the Statistical Package for Social Sciences (SSPS version 21). Continuous variables were compared with the unpaired *t*-test. Categorical variables were compared with *X*2 and *Z*-score tests. Probability variables were reported, and those less than 0.05 were considered statistically significant.

## 3. Results

Among the studied cohort, 22 HCWs (55%) were females. The mean working hours per/week were 52 ± 20 hours. The characteristics of the study population are shown in Table 1.

On the first occasion (Hand Print 0-Before HH), the hands of the 40 HCWs showed no statistically significant difference between dominant and nondominant hands as regards the type or frequency of microbial growth (Table 1). Among the transient pathogenic bacteria:* Staphylococcus aureus* was the most prevalent (38%), one-quarter of which was MRSA. Other detected pathogenic organisms were Gram-negative non-lactose fermenting (25%) and Gram-negative lactose fermenting bacteria (10%). Among Gram-negative lactose nonfermenting organisms,* Pseudomonas* constituted 0.8%. Resident flora included CoNS (19%) and anthracoids (6%) (Table 3). After alcohol hand rub (Hand Print 0-After HH), there was a significant decline in the total number of colonies detected in HCWs' handprint cultures (*p* = 0.0006). A decline was noted in all of the organisms detected in the cultures (Table 2).

We compared the Hand Print 0-After HH to Hand Print-6 weeks-After HH. The median number of bacteria/HCW hand increased but did not reach statistical significance (*p* = 0.07) (Table 3).

There was a reduction in the percent of* S. aureus* on the hands of HCWs before HH when comparing Hand Print 0-Before HH to Hand Print 12 weeks-Before HH (Table 4). On the other hand, resident commensal bacteria, namely, CoNS increased significantly (*p* < 0.0001).

### 3.1. Observation of Compliance of HCWs with HH

**Figure 3:**
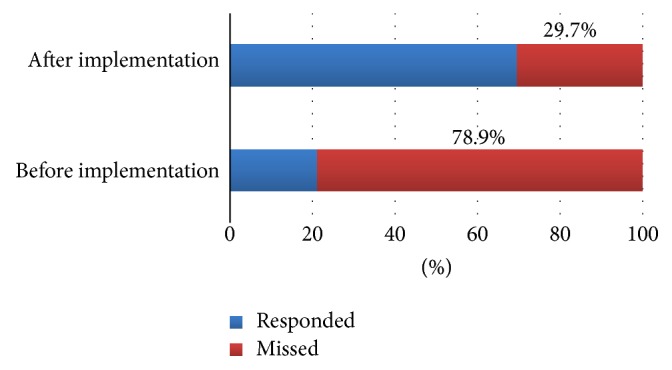
The frequency of missed opportunity before and after implementation of handprint cultures.

**Figure 4:**
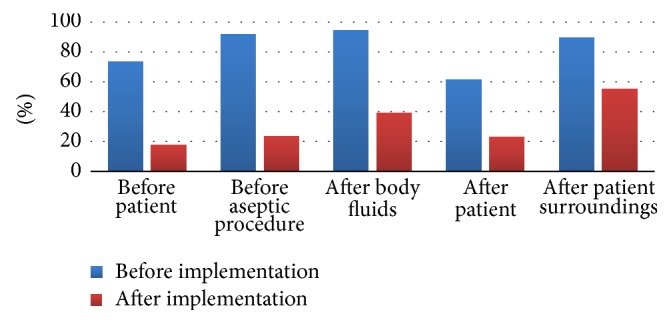
Compliance with hand hygiene before and after intervention according to the 5 moments of hand hygiene.

Before the start of this intervention, we observed 147 opportunities for HH among HCWs, 78% of the opportunities were missed. After implementation of our handprint culture training we observed another 147 opportunity for HH. There was a significant reduction in the percentage of missed opportunities (30% versus 78%, *p* < 0.0001) (Figure 3). The most frequently missed opportunity for HH, after the intervention, was after touching the patient surroundings (Figure 4).

### 3.2. Change in the Frequency of Positive Bacterial Cultures in PICUs

The results of different patients' cultures, taken at least 72 hours after admission, that is, hospital associated infections, were reviewed from the hospital database 6 months before and 6 months after intervention. Positive cultures were reduced from 51% to 37% (*p* = 0.001) (Table 2).

## 4. Discussion

Our study has shown that the use of handprint cultures before and after HH, as a visual tool, to convince the HCWs about the effectiveness of alcohol-based hand rubs on the bacterial load in the hands, could effectively increase their compliance. As observed, the missed opportunities according to “My Five Moments of HH” declined from 78% to 30% (*p* value < 0.001) with a parallel reduction in rates of positive cultures. This technique was adapted from Yamamoto et al. [8], who used this method for psychiatric hospital staff, which was effective in promoting awareness of the importance of HH and encouraged appropriate use of hand antiseptics measured by its consumption. Simple messages using appeals to social situations and to ego (self-efficacy) were rated as most likely to increase HH compliance [10].

HH using alcohol-based disinfectant could reduce the colony forming units (CFU) on hands of the HCWs. The CDC task force for HH [4] reported that the in vivo antimicrobial activity of alcohols effectively reduces bacterial counts on the hands.

In the present study, no difference was detected between the dominant and the nondominant hand on bacterial growth. In a previous study, the recovery rate of Gram-negative bacteria was higher on the nondominant hand than on the dominant hand but the difference was not statistically significant (*p* = 0.21) [11].

*Staphylococcus aureus* was the most prevalent on the hands of HCWs (38%), one-quarter of which was MRSA (almost 10% of the total). This is higher than the prevalence reported on the basis of 145 papers published between 1980 and March 2010, which was around 5%. It is assumed that MRSA rates will be higher when HCWs comply poorly with HH and contact precautions, as they are not fully aware of the threat of the bacteria load [12, 13].

The results of Hand Print-6 weeks-After HH showed a lower frequency of growth of pathogenic microorganisms compared to Hand Print 0-After HH except for the Gram-negative non-lactose fermenters. Also a third Hand Print-12 weeks-Before HH demonstrated reduction in the number of transient bacteria in the hands of HCWs compared to Hand Print 0-Before HH. The increased compliance could reduce the pathogenic microbial load on the hands of HCWs. But the CoNS increased probably because of inadequate technique.

Periodic technique simulation for the HCWs is important for the adequacy of their HH and not only their personal motivation. After a single simulation education session, critical care nurses' knowledge of and adherence to current HH guidelines remained below targeted behavior rates [14]. The effectiveness of HH as an infection control measure relates not only to the frequency with which it is carried out, but also to how effectively it is undertaken [15]. Figure 3 shows the frequency of increased compliance before and after the application of the intervention.

The most frequently missed indication was handwashing after touching patient surroundings (Figure 4). This is consistent with the results obtained from studies in different ICUs where compliance with moment 5 (after touching patient surroundings) was the lowest [16, 17]. Although it is ideal to observe all five hand hygiene moments recommended by the WHO, it is often not feasible to observe practices performed at the bedside. Therefore, some hospitals choose to observe HH before and after patient contact. It is reported that this before/after-contact monitoring method can be used as a surrogate indicator of rates based on all five moments, if certain conditions are met [18].

The rates of positive cultures in the PICU, before and after handprint culture implementation, were compared as an indirect expression of the efficacy of the intervention. It was clear that influencing the behavior of HCWs about HH by the handprint culture interview was significantly contributing factor not only in decreasing the pathogenic microbiological load on their hands but also in decreasing the rates of hospital associated infections.

## 5. Conclusion

Visual messages about the burden of bacterial growth on the hands of HCWs are important for enforcing HH and complying with “My Five Moments for HH”.

## 6. Study Limitations

Remote followup of handprint culture after several weeks to evaluate the possible Hawthorne effect during the intervention period is a limitation in the study. Another set of cultures after another 3–6 months to evaluate the burden of pathogenic bacteria in the hands of healthcare staff and to remind the healthcare staff that the value of HH on self and patient safety is needed for better patient safety.

## Figures and Tables

**Table 1:** Microbial growth over the dominant and nondominant hand of the healthcare workers before applying hand hygiene (Hand Print 0-Before HH).

CFU per type of Bacteria	Dominant hand Hand Print 0-Before HH	Nondominant hand Hand Print 0-Before HH	*p* value
Total CFU per HCW hand			
Median (min–max)	36 (0–185)	32 (0–117)	0.3
Transient bacteria			
CFU per HCW hand			
Median (min–max)	22 (2–180)	25 (1–115)	0.5
Resident bacteria			
CFU per HCW hand			
Median (min–max)	13 (2–50)	5 (1–40)	0.3

CFU= colony forming unit; HH = hand hygiene, HCW = healthcare worker.

**Table 2:** Comparison between Hand Print-0 cultures before and after applying hand hygiene.

Number of CFU according to the type of bacteria	Hand Print 0-Before HH	Hand Print 0-After HH	*p* value
Median number of CFU/dominant hand/HCW (min–max) for all bacteria	25 (1–185)	2 (1–50)	0.0006
Total number of CFU/dominant hands of the 40 HCWs	1444	134	0.003
Transient bacteria/HCWs; median number of CFU/dominant hand/HCWs (%)			
*Staphylococcus aureus*; *N* (%)	560 (38.7)	88 (65)	0.0001
Gram negative non-lactose fermenters; *N* (%)	364 (25)	22 (16)	0.0001
Gram negative lactose fermenters; *N* (%)	147 (10)	7 (5)	0.0001
*Pseudomonas*; *N* (%)	12 (0.8)	3 (2.24)	0.0001

**Table 3:** Comparison of results of Hand Print 0-After HH and Hand Print-6 weeks-After HH.

CFU according to microorganisms	Hand Print 0-After HH	Hand Print-6 weeks-After HH	*p* value
Median number of CFU/dominant hand/HCW (min–max) for all bacteria	2 (1–51)	5 (1–110)	0.07
Total number of CFU/dominant hands of the 40 HCWs	134	346	0.608
Transient bacteria/HCWs; median number of CFU/dominant hand/HCWs (%)			
*Staphylococcus aureus*; *N* (%)	88 (65.7)	155 (35)	<0.0001
Gram negative non-lactose fermenters; *N* (%)	22 (16)	75 (17)	0.13622
Gram negative lactose fermenters; *N* (%)	7 (5)	0	<0.0001
*Pseudomonas*; *N* (%)	3 (2.24)	0	0.0251
Resident bacteria/HCWs; median number of CFU/dominant hand/HCWs (%)			
Staph. CoNS; *N* (%)	12 ( 9 )	197 (45)	<0.0001
Anthracoids; *N* (%)	2 (1.5)	9 (2)	<0.0001

**Table 4:** Comparison between Hand Print 0-Before HH and Hand Print-12 weeks-Before HH.

CFU per type of Bacteria	Hand Print 0-Before HH	Hand Print-12 weeks-Before HH	*p* value
Median number of CFU/dominant hand/HCW (min–max) for all bacteria	25 (1–185)	20 (0–200)	0.66
Total number of CFU/dominant hands of the 40 HCWs	1444	1915	0.822
Transient bacteria/HCWs; median number of CFU/dominant hand/HCWs (%)			
*Staphylococcus aureus*; *N* (%)	560 (38.7)	275 (18)	<0.000
Gram negative non-lactose fermenters; *N* (%)	364 (25)	173 (9)	<0.001
Gram negative lactose fermenters; *N* (%)	147 (10)	19 (0.99)	<0.0001
Resident bacteria/HCWs; median number of CFU/dominant hand/HCWs (%)			
Staph. CoNS; *N* (%)	277 (19)	963 (50)	<0.0001
Anthracoids; *N* (%)	89 (6)	64 (4)	0.603
